# Risk factors predict post-traumatic stress disorder differently in men and women

**DOI:** 10.1186/1744-859X-7-24

**Published:** 2008-11-18

**Authors:** Dorte M Christiansen, Ask Elklit

**Affiliations:** 1 Department of Psychology, University of Aarhus, Aarhus, Denmark

## Abstract

**Background:**

About twice as many women as men develop post-traumatic stress disorder (PTSD), even though men as a group are exposed to more traumatic events. Exposure to different trauma types does not sufficiently explain why women are more vulnerable.

**Methods:**

The present work examines the effect of age, previous trauma, negative affectivity (NA), anxiety, depression, persistent dissociation, and social support on PTSD separately in men and women. Subjects were exposed to either a series of explosions in a firework factory near a residential area or to a high school stabbing incident.

**Results:**

Some gender differences were found in the predictive power of well known risk factors for PTSD. Anxiety predicted PTSD in men, but not in women, whereas the opposite was found for depression. Dissociation was a better predictor for PTSD in women than in men in the explosion sample but not in the stabbing sample. Initially, NA predicted PTSD better in women than men in the explosion sample, but when compared only to other significant risk factors, it significantly predicted PTSD for both men and women in both studies. Previous traumatic events and age did not significantly predict PTSD in either gender.

**Conclusion:**

Gender differences in the predictive value of social support on PTSD appear to be very complex, and no clear conclusions can be made based on the two studies included in this article.

## Background

It is a well established fact that women develop post-traumatic stress disorder (PTSD) more often than men do [1-3] despite the fact that men experience up to four times as many potentially traumatic events during their lifetime [3]. Though it has been suggested that the difference is mainly due to women being victims of the more toxic types of trauma, such as rape and childhood sexual abuse, women still develop PTSD twice as often as men, even when type of trauma is controlled for [2,4,5].

The sex differences related to PTSD appear to be cross-culturally consistent, though there do appear to be some cultural variations as to how pronounced they are [6]. It is therefore most likely that social gender as well as biological sex is important in making up these differences. However, as the focus of this article is not to what extent such differences are due to biological or cultural causes, we will use the terms sex differences and gender differences interchangeably.

It has been suggested that there may be more than one pathway to PTSD [7]. Saxe *et al*. [8] studied child burn victims and found that there are two separate pathways leading to PTSD: an anxiety pathway and a dissociation pathway [8]. These two pathways are separated by different risk factors, suggesting that different biobehavioural systems contribute to PTSD. The anxiety pathway may be related to the fight-or-flight system, whereas the dissociation pathway has been connected to the animal "freeze" response. Another study focusing on sexually abused children also revealed the existence of an avoidance pathway, which was more pronounced in boys than in girls [9].

To our knowledge, the existence of different pathways to PTSD has not been studied in adult samples. However, many articles focusing on gender differences have shown that men and women have different ways of responding to danger and expressing distress [3]. It has been suggested that whereas males react to stress with the well known fight-or-flight system regulated by the sympathetic nervous system, evolutionary demands has favoured an alternative tend-and-befriend system in women in times of threat [10]. The need of such a system in women is assumed to have arisen because it has not been adaptive for pregnant women or women caring for babies to run or fight in the face of danger. Instead, evolutionary adaptive behaviour has been tending to offspring, calming children down and getting them out of harm's way, and seeking protection among other members of the group. In support of this hypothesis it has been documented that whereas men generally respond to traumatic events with physiological hyperarousal and an increase in aggressive behaviours, women tend to group together and seek social support – especially from other women [10]. Women also use more dissociative mechanisms, which are mainly a passive form of defence [11]. In fact, high levels of dissociation appear to be related to the suppression of autonomic physiological responses consistent with a downregulation of both the sympathetic and HPA response to stress [11]. Therefore, even though the fight-or-flight system exists in females, the tend-and-befriend system, which is hypothesised to be regulated by the parasympathetic nervous system, is assumed to dominate in times of danger.

It thus appears that men and women respond differently to stress and though it has not been documented in adults, this may cause them to follow different pathways to PTSD. Following this line of thought, it is therefore possible that PTSD in men and women are mediated by different risk factors. Most studies do not look at gender differences when searching for risk factors predicting PTSD, but few such differences have been found. Below, we will look at gender differences in some of the risk factors related to PTSD.

### Pre-traumatic risk factors

#### Age

The mean age of onset for PTSD has been shown to differ in men and women. Hapke *et al*. found that the mean age of onset for women was 22 years, whereas for men it was 30 [12]. This could mean that increased age is a bigger risk factor for men than it is for women. Bromet *et al*. found that younger age significantly predicted PTSD in women whereas this was not the case for men [13]. However, this effect lost its significance after controlling for trauma type.

#### Previous trauma

Studies on the effects of PTSD in children have suggested that males appear to be more sensitive than females towards the devastating effects of PTSD on the developing brain [14,15], resulting in a relatively decreased resilience towards new stressors in adult male survivors of child abuse compared to female survivors [15]. Therefore, a higher correlation between PTSD and previous trauma should be expected in men compared to women. Such a gender difference has been found in two studies [1,16].

#### Anxiety and depression

Women suffer from both anxiety disorders and depression more often than men do [17]. Though anxiety and depression may be said to have some constructional overlap with PTSD, they are distinct disorders. Both anxiety and depression are well established risk factors for PTSD, but there is some evidence that anxiety and depression do not predict PTSD equally in men and women. In terms of family history of mental disorders, conflicting evidence has been found, with one study reporting a significant relationship in men, but not in women, [13] and another study reporting the opposite [3]. Regarding previous mental disorder, one study has found that pre-existing affective disorder significantly predicted PTSD in women, but not in men, whereas pre-existing anxiety disorder predicted PTSD in men, but not in women [13]

#### Negative affectivity

The overlapping constructs of negative affectivity (NA) and neuroticism are included in many factor models of personality including Costa and McCrae's five factor model of temperament where neuroticism is defined as the propensity to experience a wide variety of somatic and emotional dysphoric states including depression, anxiety, anger, and somatic symptoms [18]. People high on neuroticism are much more sensitive to stressful life events than people low on neuroticism [19], and neuroticism and NA have been shown to play a role in the development of PTSD [20,21] as well as in other psychiatric disorders [19,22].

Women tend to score higher than men on measures of neuroticism [22] and NA [23], but to our knowledge no study has examined the effects of NA/neuroticism on PTSD separately in men and women. However, gender differences have been found in how NA influences reporting of somatic symptoms [24] and depression [23], with NA being more related to symptomatology in women than in men.

### Post-traumatic risk factors

#### Persistent dissociation

Persistent dissociation is an important risk factor in PTSD [25], and some studies have found it to be a better predictor of PTSD than peritraumatic dissociation [26]. Bryant and Harvey [27] found that an initial diagnosis of acute stress disorder (ASD), which is very much based on the presence of dissociation, is a more accurate predictor of PTSD in women than in men. They concluded that the gender differences in ASD were due to gender differences in the prevalence and predictive value of persistent dissociation.

#### Social support

There is a tendency for women to report more positive support than men following traumatic events [27]. Ahern *et al*. [16] found that gender mediates the relationship between social support and PTSD so that social support has a greater protective power over women than over men, and Andrews *et al*. [28] found that the beneficial effect of positive support as well as the devastating effect of negative social attention is greater in women than in men. However, Farhood *et al*. [29] found that in Lebanese families who had been exposed to war, social support was a stronger protective factor for men than for women.

In the present article, we wish to test the hypothesis that there are gender differences in the predictive power of well established PTSD risk factors. More specifically: previous trauma, older age, and anxiety level are expected to be more predictive of PTSD in men, whereas younger age, depression, dissociation, social support, and perhaps negative affectivity are expected to be more predictive of PTSD in women. The data has been taken from two large Danish studies. The first is from an explosion in a fireworks factory and the other is from a stabbing incident at a party in a high school.

## Methods

### Sample

#### Explosion study

On the afternoon of 3 November 2004, a series of explosions hit a firework factory in a suburb of the Danish city of Kolding. A fireman was killed, about 6 residents were injured and 261 homes were partly or completely destroyed. The explosion measured 2.2 on the Richter scale and the costs of the disaster exceeded 100 million Euros. Most of the residents of the area were evacuated and many were unable to contact family members and make sure that they were safe. On average people contacted their families after 2.5 h but in one case family members were unable to contact each other for 3 days. In all, 51% of the sample had their homes either partially or completely destroyed by the explosions. Those who still had a home returned after an average of 4.5 days. For further information see Elklit [30].

#### Stabbing incident

On 3 March 2006 a young female student was stabbed to death in front of about 100 of her fellow students at a high school party in the Danish city of Aalborg. The perpetrator, a recent ex-boyfriend, later hung himself in a shed near his home. It took a very long time for most of the witnesses to realise what was happening and so the vast majority did not try to intervene. Though not all students witnessed the event, many saw the dead body inside the school through the glass doors of the entrance. Over the following weeks the students were offered counselling.

### Procedures

#### Explosion study

PTSD and a number of other variables were measured at two time points. The first (T_1_) was 3 months after the accident and the second (T_2_) was 1 year later. The procedural details have been given previously [30].

A total of 516 people (51% women, 49% men) participated in the study at T_1_. Ages ranged from 18 to 95 years with a mean age of 50.2 years (standard deviation (SD) = 14.7). The data in the present study are from the 149 participants who answered all the questionnaires at both T_1 _and T_2_. The sample that participated at both time points had a significantly higher HTQ total score than those who only participated at T_1 _(53.94 vs 50.06, F = 8.8, p ≤ 0.005). Furthermore, the participants in the follow-up sample were less likely to live alone, to be unemployed, and to have returned home after 3 months but more likely to live further away from the factory, to have been separated from their families at the time of the disaster, to have more damage to their homes and personal belongings, to have had more contact with others in a similar situation, to have received more practical help, and to have less trouble functioning. The two samples did not differ according to gender, age, education, number of children, trust in the authorities, or to which part of the disaster and its consequences that had been most disturbing.

#### Stabbing incident

The data were gathered 7 months after the incident. Questionnaires were handed out to the students still attending the school and sent to the parents' addresses of those students who had graduated in June. A total of 415 students attended the high school and 320 (77%) returned the questionnaires; 199 respondents were female (62.2%) and 121 were male. The majority of the students lived with both their parents who were generally well educated. Further data has been published elsewhere [31].

### Measures

The Harvard Trauma Questionnaire part IV (HTQ) [32] measures PTSD severity and estimates PTSD diagnosis according to the Diagnostic and Statistical Manual of Mental Disorders, version 4 (DSM-IV). The HTQ contains 32 items based on the 3 subscales of PTSD concerning a potentially distressing event. The answers are scored on a four-point Likert scale (1, "not at all"; 2, "a little"; 3, "quite a bit; 4, "all the time"). Possible total HTQ scores are in the range of 0–128, and the highest possible scores for the 3 subscales are 20 (re-experiencing), 28 (avoidance), and 20 (arousal). The HTQ part IV has been used extensively in Denmark [33], and good internal consistency, test-retest reliability and concurrent validity have been reported [32]. The alpha value for the total HTQ score was 0.93 in both the explosion and the stabbing study.

The 26-point Trauma Symptom Checklist (TSC-26) [34] has three subscales relating to negative affectivity, somatisation and dissociation. Items are rated on a four-point Likert scale ("no", "yes – sometimes", "yes – often", "very often"). The TSC generally has good reliability and good factor and criteria validity [34]. Only the subscales for NA and dissociation were used in this study. The possible score range was 0–52 for NA and 0–12 for dissociation. The alpha values in the explosion and the stabbing study were 0.85 vs 0.83 for NA and 0.63 vs 0.70 for dissociation.

The 30-question General Health Questionnaire (GHQ-30) (only data from the explosion sample) is based on the original 60-item edition of the GHQ [35]. In GHQ-30 the somatic subscale from the original GHQ has been removed and the items have been reduced to 30. The GHQ-30 therefore measures mainly psychological and psychosocial symptoms spread across five subscales measuring anxiety, feeling incompetent, depression, social dysfunction, and coping failure [36]. Items are rated on a 4-point Likert scale ("a lot worse than usual", "worse than usual", "same as usual", "better than usual"). The sensitivity and specificity of the GHQ-30 is estimated to be 81% and 80%, respectively [35]. Only the depression and the anxiety subscales were used in this study. The possible score range was 0–20 for the depression and 0–32 for the anxiety subscale. The alpha values for the two GHQ-30 subscales in the explosion study was 0.83 for depression and 0.91 for anxiety.

The Crisis Support Scale (CSS) is used for measuring perceived social support after a traumatic event [37]. The items include (1) perceived ability for someone listening, (2) contact with people in a similar situation, (3) the ability to express oneself, (4) received sympathy and support, (5) practical support, (6) the experience of being let down, and (7) general satisfaction with social support. The items are rated on a 7-point Likert scale rating from "never" to "always". Possible score range is 0–7 for each of the CSS items and 0–49 for total score. The CSS has good internal consistency and discriminatory power as well as good psychometrical reliability and validity [38]. The alpha value of the CSS was 0.70 in the explosion study and 0.73 in the stabbing study. As differences have been found regarding the ability of the different CSS items to predict PTSD, we chose to look at the different kinds of social support individually in this study. However, for ease of comparison with other studies, CSS total scores were also examined.

Previous traumatic experiences were measured by asking participants whether they had ever experienced either of the 13 different trauma types suggested by Kessler *et al*. [39]. The items were summed to establish degree of previous traumatisation.

### Statistics

In the explosion sample, PTSD measures from T_2 _were used while the independent measures were taken at T_1_. All the measures from the stabbing study were taken at the same time point.

The mean and SD are given for all measures. Pearson correlations were used to establish the direction of relationships between PTSD and independent measures. Multiple linear regression analyses were used to assess the predictive values of the different independent variables on total HTQ score. All the variables were entered into separate analyses for men and women. When the predictive value of each measure had been established the significant values for each gender were entered into a new regression analysis in order to establish which values were still significant. A 5% cut-off was used to establish significance.

## Results

### Explosion study

At T_2 _14.2% (n = 23) of the residents (6.9% of the men and 20.0% of the women, χ^2 ^= 5.60, p ≤ 0.05) suffered from PTSD and an additional 23.5% (n = 38) suffered from subclinical PTSD, missing only 1 symptom in having a full PTSD diagnosis. Women had significantly higher total HTQ scores than men and they also scored significantly higher on intrusion and arousal (all F values ≥ 6.9, all p values ≤ 0.01) but not on avoidance. The total HTQ score was 48.0 (SD = 12.5) for men and 54.6 (SD = 17.6) for women. On the 3 subscales the mean scores for men and women respectively were 6.6 (SD = 2.2) vs 7.6 (SD = 2.6) on intrusion, 10.2 (SD = 3.1) vs 11.4 (SD = 4.4) on avoidance and 8.9 (SD = 3.5) vs 10.9 (SD = 3.8) on arousal.

Mean and SD values for men and women in the explosion study can be seen in Table 1. Regression analyses for both genders are shown in Table 2.

**Table 1:** Comparison of the two trauma samples.

	**Explosion study**			**Stabbing incident**		
	**Men**	**Women**	**Total**	**Men**	**Women**	**Total**
PTSD prevalence	6.9%	20.0%	14.2%*	1.8%	14.2%	9.5%***
HTQ total	48.03 (12.55)	54.55 (17.63)	51.56 (15.80)*	46.17 (9.61)	57.16 (16.08)	52.96 (14.93)***
HTQ re-experiencing	6.64 (2.17)	7.64 (2.69)	7.19 (2.64)**	7.64 (2.37)	10.08 (3.57)	9.15 (3.38)***
HTQ avoidance	10.21 (3.08)	11.37 (4.41)	10.86 (3.91)	9.97 (2.77)	11.96 (3.63)	11.20 (3.46)***
HTQ arousal	8.88 (3.46)	10.89 (3.81)	9.99 (3.79)***	7.97 (2.94)	10.24 (3.82)	9.38 (3.68)***
Age	50.75 (15.30)	49.68 (14.05)	50.20 (14.67)	17.93 (0.98)	18.02 (1.09)	17.99 (1.05)
Previous trauma	1.65 (1.41)	1.52 (1.29)	1.58 (1.35)	1.66 (1.53)	1.54 (1.50)	1.58 (1.51)
CSS total	38.27 (5.93)	39.45 (5.93)	38.87 (5.95)*	40.50 (5.18)	40.00 (6.03)	40.19 (5.73)
CSS 1	5.94 (1.23)	6.20 (1.08)	6.07 (1.16)*	6.42 (0.98)	1.07 (0.76)	1.04 (0.06)
CSS 2	5.18 (1.36)	5.33 (1.36)	5.26 (1.36)	5.51 (1.42)	5.83 (1.45)	5.71 (1.45)
CSS 3	5.71 (1.30)	6.02 (1.20)	5.87 (1.26)**	5.38 (1.45)	5.21 (1.49)	5.28 (1.48)
CSS 4	5.90 (1.21)	5.86 (1.11)	5.88 (1.16)	6.46 (0.81)	6.26 (1.14)	6.34 (1.03)
CSS 5	4.68 (1.85)	5.06 (1.92)	4.88 (1.89)*	4.03 (2.22)	4.52 (2.01)	4.34 (2.10)
CSS 6	2.39 (1.62)	2.62 (1.81)	2.51 (1.72)	1.69 (1.37)	2.21 (1.60)	2.02 (1.54)**
CSS 7	5.19 (1.66)	5.59 (1.53)	5.39 (1.61)**	6.19 (1.06)	6.07 (1.10)	6.11 (1.09)
GHQ anxiety	15.69 (4.73)	17.33 (5.34)	16.52 (5.11)***	-	-	-
GHQ depression	7.58 (2.16)	8.02 (2.64)	7.80 (2.43)*	-	-	-
TSC NA	12.50 (2.77)	14.53 (4.35)	13.53 (3.79)***	13.06 (3.23)	15.55 (4.12)	14.6 (3.99)***
TSC dissociation	5.84 (1.45)	6.26 (1.66)	6.05 (1.57)**	6.06 (1.36)	6.81 (2.17)	6.53 (1.94)***

Means and standard deviations (SD) for the two studies for men, women, and total sample are shown

* p ≤ 0.05; ** p ≤ 0.01; *** p ≤ 0.001.

CSS, Crisis Support Scale (items include: 1, perceived ability for someone listening; 2, contact with people in a similar situation; 3, the ability to express oneself; 4, received sympathy and support; 5, practical support; 6, the experience of being let down; 7, general satisfaction with social support); GHQ-30, 30-question General Health Questionnaire; HTQ, Harvard Trauma Questionnaire part IV; NA, negative affectivity; PTSD, post-traumatic stress disorder; TSC, 26-item Trauma Symptom Checklist (only the subscales for NA and dissociation were used in this study).

**Table 2:** Regression analyses for the explosion study.

		**Men**					**Women**				
		**Beta**	**t**	**Significance**	**Adjusted R**^**2**^	**F**	**Beta**	**t**	**Significance**	**Adjusted R**^**2**^	**F**
1	(Constant)		7.31	0.0005	-0.016	0.02		6.79	0.0005	-0.007	0.511
	Age	0.02	0.14	0.89			-0.08	-0.72	0.48		
2	(Constant)		6.33	0.0005	0.014	1.45		5.74	0.0005	0.037	2.424
	Age	0.02	0.19	0.85			-0.07	-0.59	0.56		
	Previous trauma	0.21	1.69	0.10			0.24	2.08	0.04		
3	(Constant)		2.02	0.05	0.200	2.78		3.36	0.001	0.278	4.21
	Age	0.14	1.18	0.24			-0.08	-0.80	0.42		
	Previous trauma	0.06	0.48	0.63			0.24	2.40	0.02		
	CSS 1	0.06	0.42	0.68			0.24	1.70	0.10		
	CSS 2	0.12	0.97	0.34			0.01	0.06	0.95		
	CSS 3	-0.14	-1.15	0.26			-0.27	-2.63	0.01		
	CSS 4	0.06	0.44	0.66			-0.34	-2.51	0.02		
	CSS 5	-0.05	-0.47	0.64			-0.01	-0.05	0.96		
	CSS 6	0.49	3.78	0.0005			0.28	3.15	0.002		
	CSS 7	-0.13	-0.98	0.33			0.25	1.68	0.10		
4	(Constant)		0.64	0.52	0.594	9.51		1.18	0.24	0.418	5.90
	Age	0.08	0.94	0.35			-0.07	-0.76	0.45		
	Previous trauma	0.19	2.12	0.04			0.17	1.88	0.07		
	CSS 1	0.05	0.52	0.61			0.22	1.70	0.09		
	CSS 2	-0.04	-0.50	0.62			0.01	0.08	0.94		
	CSS 3	-0.18	-2.04	0.05			-0.22	-2.25	0.03		
	CSS 4	0.19	1.93	0.06			-0.15	-1.15	0.26		
	CSS 5	-0.00	-0.02	0.99			-0.03	-0.26	0.80		
	CSS 6	0.25	2.56	0.01			0.24	2.12	0.04		
	CSS 7	-0.06	-0.63	0.53			0.15	1.11	0.27		
	GHQ-30 anxiety	0.75	6.41	0.0005			0.17	1.13	0.26		
	GHQ-30 depression	-0.11	-0.98	0.33			0.32	2.37	0.02		
5	(Constant)		-0.01	0.99	0.662	10.65		-0.34	0.73	0.598	9.58
	Age	0.11	1.30	0.20			0.04	0.44	0.66		
	Previous trauma	0.12	1.37	0.18			0.11	1.43	0.16		
	CSS 1	0.04	0.44	0.66			0.16	1.48	0.14		
	CSS 2	-0.06	-0.68	0.50			0.07	0.72	0.48		
	CSS 3	-0.17	-2.07	0.04			-0.12	-1.39	0.17		
	CSS 4	0.21	2.18	0.03			-0.20	-1.74	0.09		
	CSS 5	-0.01	-0.08	0.93			0.06	0.73	0.47		
	CSS 6	0.18	1.98	0.053			0.16	1.61	0.11		
	CSS 7	-0.08	-0.88	0.38			0.17	1.49	0.14		
	GHQ-30 anxiety	0.53	4.13	0.0005			-0.14	-0.10	0.32		
	GHQ-30 depression	-0.16	-1.59	0.12			0.10	0.87	0.39		
	TSC-26 NA	0.27	1.73	0.09			0.48	3.71	0.0005		
	TSC-26 dissociation	0.15	1.26	0.21			0.27	2.37	0.02		

Beta values, t values, significance, adjusted R^2^, and F values for men and women following the explosions at the firework factory are shown.

CSS, Crisis Support Scale (items include: 1, perceived ability for someone listening; 2, contact with people in a similar situation; 3, the ability to express oneself; 4, received sympathy and support; 5, practical support; 6, the experience of being let down; 7, general satisfaction with social support); GHQ-30, 30-question General Health Questionnaire; NA, negative affectivity; TSC, 26-item Trauma Symptom Checklist (only the subscales for NA and dissociation were used in this study).

#### Age

Ages ranged from 18 to 95 years. For men the mean age was 50.8 years (SD = 15.3) and for women it was 49.7 years (SD = 14.1). Age did not correlate significantly with degree of PTSD and did not significantly predict PTSD severity in the regression analyses for either gender.

#### Previous trauma

Men had experienced more different traumatic events than women with an average of 1.7 (SD = 1.4) compared to 1.5 (SD = 1.3). However, this difference was not significant. The number of previously experienced trauma types correlated significantly with PTSD severity (r = 0.21, p ≤ 0.001). Previous trauma did not significantly predict PTSD symptomatology in men but it did reach significance in the regression analysis for women at step two and three, until depression and anxiety were introduced.

#### Social support

The mean total score on the CSS was 38.3 (SD = 6.0) for men and 39.5 (SD = 5.6) for women. This difference was significant (F = 4.89, p ≤ 0.05). Total CSS score had a moderate negative correlation with PTSD severity (r = -0.22, p ≤ 0.006). Three items correlated significantly with degree of PTSD. For ability to express oneself (r = -0.18, p < 0.0005) and received sympathy and support (r = -0.30, p < 0.0005) the correlation was moderate and negative, whereas experiencing being let down had a moderate and positive correlation with PTSD severity (r = 0.37, p < 0.0005).

For men, total CSS score significantly predicted PTSD severity until depression and anxiety were controlled for. When the different items of the CSS were entered into the regression analysis together, only the experience of being let down gained significance (p ≤ 0.0005). However, at the final level of analysis the ability to express oneself and received sympathy and support significantly predicted PTSD severity, while the experience of being let down was only almost significant (p = 0.053).

In women, the total CSS score was significant when entered at step three but not after that. When the different CSS items were entered separately into the analysis the ability to express oneself, received sympathy and support, and feeling let down were all significant. However none of them remained so when NA and dissociation were controlled for.

#### Anxiety

The mean anxiety score on the GHQ-30 anxiety subscale was 15.7 (SD = 4.8) for men and 17.3 (SD = 5.3) for women. The mean score for women was significantly higher than for men (F = 13.12, p ≤ 0.0005). Anxiety correlated highly (r = 0.60, p ≤ 0.0005) with PTSD severity and significantly predicted PTSD symptoms in men (p ≤ 0.0005), even at the final level of analysis. However, it did not reach significance for women.

#### Depression

The mean depressive score for men measured by the depressive subscale of the GHQ-30 was 7.6 (SD = 2.2) and for women it was 8.0 (SD = 2.6). This difference was significant (F = 4.09, p ≤ 0.05). Depression correlated significantly with total HTQ score (r = 0.48, p ≤ 0.0005). Depression did not significantly predict PTSD severity in men but it did reach significance in women until NA and dissociation were controlled for.

#### Negative affectivity

The mean NA score measured by the TSC-26 was 12.5 (SD = 2.8) for men, which was significantly lower (F = 38.0, p ≤ 0.001) than the mean score for women of 14.5 (SD = 4.3). NA correlated significantly with PTSD severity (r = 0.70, p ≤ 0.0005) and significantly predicted PTSD severity in women, but not in men when introduced at the final level of analysis.

#### Dissociation

The mean score on the dissociative TSC-26 subscale was 5.8 (SD = 1.5) for men and 6.3 (SD = 1.7) for women. The difference was significant (F = 9.15, p ≤ 0.05). Dissociation and the total HTQ score had a high and significant correlation (r = 0.63, p ≤ 0.0005). Dissociation was not even close to reaching significance in the male model, but for women it significantly predicted PTSD severity even at the final level (p ≤ 0.05).

### Significant risk factors

The next step was putting the significant risk factors into new regression analyses to see how much of the variance they could explain. For men, the original model explained 66% of the PTSD variance. Experiencing being let down, anxiety, and NA were entered into a second regression analysis. Though feeling let down and NA did not reach significance at step five of the original model, they were considered close enough to be included in this final analysis. The CSS items that did not reach significance when first introduced into the original analysis were not included. The three variables were all significant and together they explained 65% (F = 42.47) of the PTSD variance in men.

For women, the measures closest to remaining significant in the original model again failed to remain significant when NA and dissociation were controlled for. NA and dissociation were both highly significant (p ≤ 0.0005) and explained 54% (F = 46.71) of the PTSD variance compared to the 60% explained by the original model.

#### Stabbing incident

At 7 months after the stabbing incident 28 students (9.5%; 1.8% of the men and 14.2% of the women) met the DSM-IV criteria for PTSD. This difference was significant (χ^2 ^= 12.6, p ≤ 0.0005). An additional 25.1% (n = 74) could be diagnosed with subclinical PTSD, meeting full criteria for only two of the three symptom clusters. The mean total HTQ score was 57.2 (SD = 16.1) for women and 46.2 (SD = 9.6) for men. The mean scores for men and women respectively on the subscales were 7.6 (SD = 2.4) vs 10.1 (SD = 3.6) on intrusion, 10.0 (SD = 2.8) vs 12.0 (SD = 3.7) on avoidance, and 8.0 (SD = 3.0) vs 10.2 (SD = 3.8) on arousal. Women scored significantly higher on total HTQ as well as on each of the three symptom scales (all F values > 2.25, all p values ≤ 0.0005).

Mean and SD values for men and women in the stabbing sample are shown in Table 1. The two original separate regression analyses are shown in Table 3.

**Table 3:** Regression analyses for the stabbing incident.

		**Men**					**Women**				
		**Beta**	**t**	**Significance**	**Adjusted R**^**2**^	**F**	**Beta**	**t**	**Significance**	**Adjusted R**^**2**^	**F**
1	(Constant)		3.06	0.003	-0.010	0.10		1.73	0.09	0.001	1.09
	Age	-0.03	-0.31	0.76			0.08	1.04	0.30		
2	(Constant)		3.18	0.002	0.059	3.98		1.25	0.21	0.033	3.74
	Age	-0.05	-0.50	0.62			0.11	1.37	0.17		
	Previous trauma	0.28	2.80	0.006			0.20	2.51	0.01		
3	(Constant)		3.10	0.003	0.183	3.36		2.69	0.01	0.406	13.13
	Age	-0.07	-0.671	0.50			0.12	1.85	0.07		
	Previous trauma	0.28	2.86	0.005			0.17	2.64	0.01		
	CSS 1	0.15	1.24	0.22			-0.14	-1.93	0.06		
	CSS 2	-0.03	-0.29	0.77			-0.08	-1.12	0.26		
	CSS 3	-0.16	-1.57	0.12			-0.24	-3.62	0.0005		
	CSS 4	-0.06	-0.46	0.64			0.11	1.29	0.20		
	CSS 5	0.18	1.84	0.07			0.17	2.65	0.01		
	CSS 6	0.24	2.31	0.02			0.35	4.35	0.0005		
	CSS 7	-0.16	-1.31	0.19			-0.20	-2.09	0.04		
4	(Constant)		0.39	0.70	0.549	11.53		-0.19	0.85	0.777	51.73
	Age	0.02	0.23	0.82			0.05	1.24	0.22		
	Previous trauma	0.13	1.70	0.09			0.00	0.07	0.95		
	CSS 1	0.18	2.02	0.05			-0.01	-0.11	0.91		
	CSS 2	0.13	1.70	0.09			-0.03	-0.64	0.53		
	CSS 3	-0.09	-1.20	0.23			-0.13	-3.20	0.002		
	CSS 4	-0.05	-0.50	0.62			0.04	0.67	0.50		
	CSS 5	0.04	0.51	0.61			0.11	2.91	0.004		
	CSS 6	0.24	3.11	0.003			0.25	4.96	0.0005		
	CSS 7	-0.13	-1.50	0.14			-0.02	-0.29	0.77		
	TSC-26 NA	0.38	4.57	0.0005			0.44	8.93	0.0005		
	TSC-26 dissociation	0.36	4.32	0.0005			0.37	7.73	0.0005		

Beta values, t values, significance, adjusted R^2^, and F values for men and women following the high school stabbing are shown.

CSS, Crisis Support Scale (items include: 1, perceived ability for someone listening; 2, contact with people in a similar situation; 3, the ability to express oneself; 4, received sympathy and support; 5, practical support; 6, the experience of being let down; 7, general satisfaction with social support); GHQ-30, 30-question General Health Questionnaire; NA, negative affectivity; TSC, 26-item Trauma Symptom Checklist (only the subscales for NA and dissociation were used in this study).

#### Age

The students were aged 16 to 20 years. The mean age for women was 18.0 (SD = 1.1) and for men it was 17.9 (SD = 1.0). Age did not correlate significantly with degree of PTSD and it did not significantly predict PTSD severity in either gender.

#### Previous trauma

Men had experienced more traumatic events than women with an average of 1.7 traumatic events (SD = 1.5) compared to 1.5 in women (SD = 1.5). However, this difference was not significant. Previous trauma correlated significantly with total HTQ score (r = 0.20, p ≤ 0.001) and significantly predicted PTSD severity in both men and women. However, this significance was lost for both genders after controlling for NA and dissociation.

#### Social support

The mean CSS total score was 40 (SD = 5.7) for both men and women. Perceived ability for someone listening, ability to express oneself, received sympathy and support, and general satisfaction all had moderate negative correlations with PTSD severity (all r values < -0.24, all p values ≤ 0.0005) while experiencing being let down had a high and positive correlation with PTSD symptoms (r = 0.49, p ≤ 0.001).

For men, total CSS score did not significantly predict PTSD severity when introduced at step three. When the different items were entered into the regression analysis, feeling let down was the only CSS item to gain significance when first entering the analysis, and it remained so after controlling for dissociation and NA (p ≤ 0.0005). In women total CSS score was highly significant when it entered the analysis at step three and still at the final step. When the CSS items were entered into the model separately, ability to express oneself, practical support and feeling let down were significant, both when first entering and at the final level of analysis. General satisfaction with social support was significant when first introduced but not when dissociation and NA were controlled for. The perceived ability of having someone who would listen almost reached significance when first introduced.

#### Negative affectivity

The mean score for NA measured by the TSC-26 was 13.1 (SD = 3.2) for men, which was significantly lower (F = 32.1, p ≤ 0.0005) than the mean score for women of 15.5 (SD = 4.1). NA correlated highly with PTSD severity (r = 0.76, p ≤ 0.0005) and was highly significant in the regression analyses (p ≤ 0.0005) for both men and women.

#### Dissociation

The mean score on the dissociative TSC-26 subscale was 6.1 (SD = 1.4) for men and 6.8 (SD = 2.5) for women. This difference was significant (F = 11.5, p ≤ 0.001). Dissociation correlated highly with total HTQ score (r = 0.70, p ≤ 0.001) and significantly predicted PTSD symptoms in both men and women.

#### Significant risk factors

As with the explosion sample, the significant risk factors were put into a new regression analysis. The original male model explained 55% of the PTSD variance. When a new model was created based on the four factors that were significant when they were first entered into the original model, previous trauma failed to remain significant after dissociation and NA were controlled for. However, experiencing being let down, dissociation, and NA were all highly significant (p ≤ 0.0005), explaining 54% of the total variance.

In women, the original model accounted for 78% of the variance. The CSS items that reached or almost reached significance at step three were entered into a new regression analysis together with previous trauma, NA, and dissociation. At the final level of this model, ability to express oneself, practical support, feeling let down, NA, and dissociation were all significant (p ≤ 0.05) and the model explained 77% of the PTSD variance.

## Discussion

### PTSD prevalence

The PTSD prevalence of 14.2% and 9.5% in the explosion and the stabbing study, respectively, are quite high considering the time of measurement (15 and 7 months, respectively). Women had a significantly higher PTSD prevalence than men in both studies. The PTSD prevalence was somewhat higher in the explosion study than in the stabbing incident for both women (20.0% vs 14.2%) and men (6.9% vs 1.8%). The female/male PTSD ratio was somewhat higher in the stabbing sample (7:1) than in the explosion sample (3:1). Additionally, women scored significantly higher on the HTQ as well as on each of the three subscales, except for avoidance where the difference in the explosion study was not significant.

### Age

The hypothesis that higher age would increase the PTSD risk in men and decrease it in women was not supported. Age did not correlate with PTSD severity and did not predict PTSD for either gender in either sample. The lack of significance in the stabbing sample could be explained by the small range in age but this cannot explain the results in the explosion sample. Though this result is in contrast to our hypothesis, it is consistent with the finding in the Bromet *et al*. [13] study mentioned earlier, that the relationship between age and PTSD in women lost significance when trauma type was controlled for, as the two samples included in this study focused on just one trauma type each.

### Previous trauma

Contrary to what should be expected based on the age difference of the two studies, the number of different previous traumatic experiences was exactly the same in the two samples with men having on average experienced 1.7 and women 1.5 traumatic events. The gender difference in numbers of traumatic events experienced did not reach significance in either sample. Contrary to the findings by Ahern *et al*. [16] from a heavily exposed war sample, previous trauma did not predict PTSD better in men than in women. In the stabbing sample, previous trauma was significant for both genders until dissociation and NA were controlled for, whereas in the explosion sample it only reached significance for women and only until depression and anxiety were controlled for.

### Social support

Gender differences regarding the amount of positive social support received were only significant for certain kinds of support in the explosion sample, and even then the differences were not big. Women in the stabbing sample felt significantly more let down than the men did, but again, scores did not differ much across gender. In contrast to our hypothesis, social support as a whole did not predict PTSD severity better in women than in men in either sample. However, there were some gender differences regarding the predictive power of the individual CSS items, although these are not easily interpreted. In the explosion study, feeling let down was among the best predictors of PTSD in men, whereas in women it did not remain significant after controlling for dissociation and NA. Perhaps this is because NA did not predict PTSD severity in men, whereas it was highly significant for women. Interestingly, as can be seen in Table 2, ability to express oneself and received sympathy and support were both significant for women when first entering the regression analysis in the explosion sample, but not for men. Whereas the two items then lost significance for women when other variables were controlled, the same two items became significant for men later in the analysis when NA, dissociation, and especially anxiety were controlled for – suggesting that the ability to express oneself as well as received sympathy and support indirectly decreases the risk of developing PTSD by decreasing anxiety levels. This effect was not seen in women, presumably because anxiety did not reach significance. This suggests that the relationship between social support and PTSD is far from straight forward. In the stabbing sample, however, there was some support for the hypothesis. While feeling let down was the only CSS item to reach significance in men, the ability to express oneself and practical support significantly predicted PTSD symptoms in women along with being let down. Unfortunately, we were not able to control for anxiety and depression in the stabbing sample.

In both genders, dissatisfaction with support was a better predictor of PTSD levels than actual support, and even though this may to some degree be mediated by negative affectivity, feeling let down remained a significant predictor of PTSD even after NA was controlled for in all samples except for the women in the explosion study.

It is important to notice, that in both studies the subjects generally experienced good support. It is quite possible that social support would have had more discriminative power in a "less privileged" sample where the victims diverge more in the amount and quality of the support they receive. Additionally, concerning the amount of positive support received from others, gender differences were only significant in the explosion sample, and even here they were small.

It is furthermore possible that gender differences in the effect of social support on PTSD are mediated by cultural factors such as gender role. This would explain why the studies mentioned earlier have reached different conclusions as to the effect of social support on PTSD in men and women. Though the two samples studied here are from very similar backgrounds, it is possible that cultural influence on social support is mediated by age and that this can explain the different findings in the two studies.

### Anxiety

Anxiety was not measured in the stabbing sample but, as expected, women in the explosion sample scored significantly higher on the anxiety subscale of the GHQ-30 than men. In support of our hypothesis, anxiety did not significantly predict PTSD severity in women – not even before controlling for NA – but it did predict PTSD severity in men and remained significant even at the final level of analysis. This is in line with the findings by Bromet *et al*. [13]. However, it is important to notice that whereas Bromet *et al*. used a measure of pre-existing psychopathology, the GHQ in the present study measured initial levels of anxiety after the traumatic event.

### Depression

As with anxiety, depression was only assessed in the explosion study. As expected, the women in this study were significantly more depressed than the men. Depression only reached significance in predicting PTSD severity in women but not in men. This finding is in line with Bromet *et al*.'s study [13], although the two studies differ in time of measurement, as Bromet has focused on depression prior to traumatic exposure. However, depression did not remain significant in women when NA was controlled for. This is probably due to NA having a moderating effect on the relationship between depression and PTSD.

### Negative affectivity

Women scored significantly higher than men on NA in both samples. NA correlated significantly with PTSD severity in both samples. The hypothesis that NA would predict degree of PTSD better in women than in men was supported to some extent in the explosion sample but not in the stabbing sample. In the explosion sample, NA did not reach significance in the original analysis when introduced at the final level. However, when entered in the final model based on the significant or nearly significant risk factors from the original model, NA also significantly predicted PTSD in the male part of the explosion sample.

### Dissociation

Women dissociated significantly more than men in both studies, which is in line with what some studies have found for peritraumatic dissociation. The hypothesis that dissociation would predict PTSD in women, but not in men, was supported in the explosion sample, but not in the stabbing sample, where it was a highly significant predictor of PTSD severity in high school students of both genders. It is not known whether the differing results from the two studies is caused by differences in age, trauma type, time of measurement, or some other factor.

It is highly relevant to study this possible gender difference further, because if dissociation (peritraumatic or persistent) only predicts PTSD in women, the somewhat contradictory findings in the area, which are particularly evident for peritraumatic dissociation, may be due to studies being based on samples consisting of both genders.

All in all, these risk factors explain 54% of the PTSD variation for men in the stabbing sample and 77% for women. In the explosion sample, these numbers are 65% for men but only 54% for women. The high percentage in the young women is probably due to more kinds of social support being significant, whereas the relatively high percentage explained in the male explosion sample is probably due to anxiety being significant.

### Limitations

The findings in this study are based on samples from two geographical regions in Denmark and the samples were primarily made up from white, middle class Danish participants. Future research needs to examine whether the gender differences found in this study are also evident in samples with more diverse backgrounds and socioeconomic status.

We have compared results from two studies that differ from one another on a number of points. First, there is the obvious difference in trauma types. The explosion study was a devastating industrial accident, whereas the stabbing incident was an intentional, interpersonal assault. The latter trauma types usually results in a higher prevalence of PTSD but in this study, although the difference was not great, the highest prevalence of PTSD was in the explosion study. This is probably due to the high degree of destruction following the explosions, which caused a greater number of the participants to be directly affected by the trauma due to life changes, loss of home, and relocation, than was the case following the stabbing incident. It is possible that the differences in trauma types have affected some of the discrepancies in the findings of the two studies.

Second, the explosion sample had a higher mean age but also a greater age span then the stabbing sample. Whereas the explosion sample was made up of adults, most of the participants from the stabbing incident were still teenagers. This may increase the likelihood that our findings can be extrapolated to other trauma victims. However, it may also be a limitation as it is unknown whether differences in the findings in the two studies are due to the difference in age or other factors. For example, age may affect the relationship between PTSD and some of the variables (e.g. social support), thus leading to different findings in the two studies. It is also quite likely that gender differences are not as evident in a young sample, as the cultural and genetic bases for such differences may not yet be fully developed. The young age of the stabbing sample may be particularly relevant when studying NA because this is hypothesised to be a personality factor. It can be argued that it does not make sense to examine a stable trait in teenagers, some of which may be said not yet to have developed a stable personality.

Finally, the explosion study is of a longitudinal design, whereas this is not the case for the school stabbing. Where the two studies have agreed in their findings, this difference in design may be seen as a strength because it shows that the relationship between PTSD and the studied variable appears to be stable across time. However, where there are discrepancies between the findings from the two studies, it is not clear to what extent this may be due to design differences or, as stated above, to differences in trauma types, age, or unknown factors.

### Future research

Many studies have examined risk factors in PTSD in samples consisting of both men and women. The results of this study show that gender specific analyses provide more detailed information on the relationship between PTSD and suspected risk factors. We have found significant gender differences in the relationship between PTSD, depression, anxiety, social support and dissociation. Studying these factors in both-sex samples may lead to conflicting findings as has often been the case. Therefore, future studies of different trauma samples should examine the predictive power of different factors separately in men and women in order to gain a better understanding of their relationship with PTSD.

As mentioned earlier, the relationship between social support and PTSD appear to be quite complex, as it is mediated by other risk factors. This study only assessed some of the risk factors that have shown to be related to the development of PTSD, and other factors not examined here (e.g. different ways of coping with trauma) may affect the relationship between social support and PTSD. This is an important area of future research that may help to shed more light on the development of PTSD in men and women.

The purpose of this study was to test whether the same risk factors predict PTSD in men and women. If different risk factors are important for men and women it does not necessarily mean that the two genders follow different pathways to PTSD. However, had we not found any such gender differences, it would definitely speak against the different pathways hypothesis. Future studies should focus more on the possibility that men and women follow different pathways to PTSD and that this may lead to more differences in the symptomatology of the disorder than has so far been discovered. If this is in fact the case it will be worth keeping in mind when we seek out and test different kinds of treatment as there may also be gender differences in the effect of PTSD treatments in men and women, depending on which pathway led to the disorder.

### Clinical implications

Though the findings in this study need to be replicated, the potential implications for PTSD treatment are substantial. If PTSD in men and women, at least to a certain degree, are mediated by different risk factors then this may very well lead to gender differences in the course and other characteristics of the disorder. Ultimately, such gender differences may affect treatment efficacy, so that one treatment may be affective for women but not for men, or the other way around. Today, most treatment research is based on both-gender samples (with the exception of trauma specific samples such as war veterans and rape victims). The findings in this study points to a need to look at effects of treatment differentially in men and women. Thus, if any gender differences in treatment effects are found, they can influence the treatment programs that are offered to men and women with PTSD.

## Conclusion

In this article we have used data from two different studies to test the hypothesis that risk factors predict PTSD differently in men and women. The hypothesis was partially supported. For age and previous trauma our hypotheses were not supported, as these risk factors did not significantly predict PTSD in either gender. For NA, there was an initial gender difference in the explosion sample, but in the final analyses, NA significantly predicted PTSD in both men and women from both samples, which was contrary to our expectations. In support of our hypotheses, anxiety significantly predicted PTSD in men but not in women, and the reverse was true for depression, although this risk factor did not remain significant in women after NA was controlled for. However, these two measures were only assessed in the explosion sample. The hypothesis for dissociation was supported in the explosion sample but not in the younger stabbing sample. Social support was not found to be a better predictor of PTSD in women than in men. Although more kinds of social support significantly predicted PTSD in the young women of the stabbing sample, this was not the case in the explosion sample, and the most significant risk factor, the feeling of being let down, did not predict PTSD better in women than in men – actually the contrary occurred in the explosion sample. The relationships between social support and PTSD in men and women appear to be complex.

Though our results are not without contradictions this study emphasises the importance of conducting more research in this area. If different factors are important in predicting PTSD in men and women, it may result in differences in the resulting psychopathology, which in turn may result in differential treatment effects in men and women.

## Competing interests

The authors declare that they have no competing interests.

## Authors' contributions

AE carried out the studies, performed the statistical analyses, supervised the writing of the article and drafted the manuscript. DMC performed the statistical analyses and wrote the article. Both authors read and approved the final manuscript.
